# Advances and Perspectives of Transgenic Technology and Biotechnological Application in Forest Trees

**DOI:** 10.3389/fpls.2021.786328

**Published:** 2021-11-30

**Authors:** Yiyi Yin, Chun Wang, Dandan Xiao, Yanting Liang, Yanwei Wang

**Affiliations:** ^1^National Engineering Laboratory for Tree Breeding, Key Laboratory of Genetics and Breeding in Forest Trees and Ornamental Plants, Ministry of Education, The Tree and Ornamental Plant Breeding and Biotechnology Laboratory of National Forestry and Grassland Administration, Beijing Forestry University, Beijing, China; ^2^College of Biological Sciences and Biotechnology, Beijing Forestry University, Beijing, China; ^3^Beijing Advanced Innovation Center for Tree Breeding by Molecular Design, Beijing Forestry University, Beijing, China

**Keywords:** forest trees, transgenic technology, genetically modified trees, trait improvement, application

## Abstract

Transgenic technology is increasingly used in forest-tree breeding to overcome the disadvantages of traditional breeding methods, such as a long breeding cycle, complex cultivation environment, and complicated procedures. By introducing exogenous DNA, genes tightly related or contributed to ideal traits—including insect, disease, and herbicide resistance—were transferred into diverse forest trees, and genetically modified (GM) trees including poplars were cultivated. It is beneficial to develop new varieties of GM trees of high quality and promote the genetic improvement of forests. However, the low transformation efficiency has hampered the cultivation of GM trees and the identification of the molecular genetic mechanism in forest trees compared to annual herbaceous plants such as *Oryza sativa*. In this study, we reviewed advances in transgenic technology of forest trees, including the principles, advantages and disadvantages of diverse genetic transformation methods, and their application for trait improvement. The review provides insight into the establishment and improvement of genetic transformation systems for forest tree species. Challenges and perspectives pertaining to the genetic transformation of forest trees are also discussed.

## Introduction

As important plant materials, forest trees are crucial for ecological preservation, climate regulation, building materials, road greening, and energy supply (Trumbore et al., 2015). However, breeding forest trees take a long time, and advanced-generation breeding populations are needed due to their long life span. Molecular breeding methods based on genetic transformation facilitate breeding and overcome the disadvantages of directional improvement, breeding, and the difficulty of distant hybridization and inter-specific hybridization that characterizes traditional breeding (Fang and Han, 2019). Furthermore, two or more traits can be simultaneously improved in genetically modified (GM) trees, enabling the improvement of the adaptability and productivity of forest trees (Martínez-Gómez, 2019). Genetic transformation is complicated by the complexity and diversity of forest genomes. Compared to annual crops, transgenic research on forestry trees started late and efficient transformation systems for many tree species have not been successfully established. Thus, it is important and indispensable to understand the parameters of genetic transformation of forest trees and elucidate challenges in transgenic technology, accelerating the application of GM trees for sustainable development.

To date, transgenic investigation of trees has focused on the transformation efficiency using marker genes, the regeneration of the transgenic plant tissues into complete plants, the introduction of introducing exogenous genes into receptor genomes, and gene function and regulation mechanism (Yu et al., 2021). Genetic engineering has been successfully applied to forest trees including *Populus*, used as a model plant for gene function research based on the establishment of a complete genetic transformation system (Zhou Y. et al., 2020; Guo et al., 2021). To generate GM forest trees, the most widely used transgenic method involves *Agrobacterium tumefaciens*-mediated. Gene gun-mediated, pollen tube pathway, and protoplast transformation methods can also be used. Mobile genetic techniques, such as clustered regularly interspaced short palindromic repeats (CRISPR)-associated (CRISPR-Cas) systems, have recently been applied to forest tree breeding (Shivram et al., 2021). Transgenic technology has been used to modify insect, herbicide, abiotic stress, disease resistance, wood properties, flowering regulation, and phytoremediation (Liao and An, 2013; Ding et al., 2016; Xu and Zhai, 2021; Yu et al., 2021).

In this study, we reviewed the advantages and disadvantages of diverse genetic transformation methods and their application to forest trees for enhancing insect, disease, and abiotic-stress resistance. Also, the challenges in transgenic engineering of forest trees are presented, and potential future work is discussed. This review provides insight into the establishment and improvement of genetic transformation systems for forest tree species.

## Advancements in Transgenic Technologies for Forest Trees

### *Agrobacterium tumefaciens*-Mediated Transformation

*Agrobacterium tumefaciens*-mediated transformation is the most commonly used method of genetic transformation of forest trees. *A*. *tumefaciens* could deliver DNA molecules into plant cells for integration of exogenous genes into the host genome (Chilton et al., 1977). *A*. *tumefaciens*-mediated genetic transformation of plants is a rare example of naturally occurring trans-kingdom DNA transfer (Lacroix and Citovsky, 2013). *A*. *tumefaciens*-mediated genetic transformation system has been widely applied in poplars, such as *Populus tomentosa*, *Populus alba* × *Populus glandulosa*, *Populus simonii* × *Populus nigra*, and *Cinnamomum camphora* (Table 1) (Du et al., 2008; Li et al., 2020a,b; Ma et al., 2020; Guo et al., 2021). Furthermore, it has also been successfully established in some other forest trees, such as *Betula platyphylla*, *Eucalyptus urophylla*, and *Juglans* (Wang, 2015; Li et al., 2021a; Zhong et al., 2021).

**TABLE 1:** Transgenic plants successfully obtained by *Agrobacterium tumefaciens* transformation.

Tree species	Trait	Gene	Transformation receptor	References
*P. tremula* × *P. alba*	Wood material improvement	*CCoAOMT*	Leaf disk/stem	Meyermans et al., 2000
*P. deltoides* × *P. simonii*	Insect-resistant	*Bt*	Stem	Rao et al., 2000
*P. deltoides* × *P. simonii*	Insect-resistant	*AaIT*	Leaf disk/stalk	Wu et al., 2000
*P. tremula* × *P. alba*	Herbicide resistance	*gsh I*	Leaf disk	Gullner et al., 2001
*Juglans nigra*	Antimicrobial properties	*ThEn-4*	Somatic embryo	Tang et al., 2001
*Betula platyphylla*	Insect-resistant	Insecticidal peptide gene of spider; NptII; *GUS*	Leaf disk; stem; stalk	Zhan et al., 2001
*P. tremula* × *P. tremuloides*	Insect-resistant	*Cry3Aa*	Stem	Génissel et al., 2003
*P. tremuloides*	Wood material improvement	*Pt4CL*;	Leaf disk	Li et al., 2003
		*LsCAld5H*		
*P. pseudocerasus*	Antimicrobial properties	*Cecropin B/Shiva A*	Stem tip	Wang et al., 2003
*P. deltoides*	Disease resistance	*CH5B*	Leaf disk	Meng et al., 2004
*triploid P. tomentosa*	Lignin	Antisense *CCoAOMT*	Seedling	Zhao et al., 2004
*P. tomentosa* Carr	Insect-resistant	*Cry1Ac*; *API*	Leaf disk	Li et al., 2007
*Cinnamomum camphora*	Selection markers	*GUS/GFP*	Embriotic callus	Du et al., 2008
*P. tremula* × *P. tremuloides*	Herbicide resistance	*BARNASE*;	leaf disc/stem	Li et al., 2008
*P. tremula* × *P. alba*		*BAR*		
*P. tremula* × *P. alba;*	Disease resistance	*PtWRKY23*	Leaf disk	Levee et al., 2009
*P. nigra* × *P. maximowiczii*				
*P. tremula* × *P. alba*	Wood material improvement	*GS1a*	Stem	Coleman et al., 2012
*P. tomentosa* Carr.	Disease resistance	*Bbchit1*;	Leaf disk	Huang et al., 2012
		*LJAMP2*		
*P. ciliata* Wall.	Wood material improvement	*anti-CAD*	Stalk	Thakur et al., 2012
*P.* .*euramericana* cv.	Cell wall remodeling	*PtrMAN6*	Leaf disk	Zhao et al., 2013
*P. alba* × *P. tremula var. glandulosa*	Wood material improvement	*PdGA20ox1*	Stem	Park et al., 2015
*P. davidiana* × *P. bolleana*	Salt tolerance	*PtSOS2*	Leaf disk	Yang et al., 2015
*P. alba* × *P. tremula var. glandulosa*,	Woody biomass	*PdGA20ox1*	Leaf disk	Jeon et al., 2016
*P. alba* × *P. glandulosa*	Drought resistance, salt and cold tolerance	*codA*	Shoots	Ke et al., 2016
*P. simonii* × *P. nigra*	Salt tolerance	*ERF76*	Twigs	Yao et al., 2016
*P. euramericana*	Insect resistant, salt tolerance	*Cry1Ac, Cry3A, BADH*	Leaves	Yang et al., 2016
*P. davidiana* × *P. bolleana*	Insect resistant	*Cry1Ac* + *SCK, Cry1Ah3, Cry9Aa3*	Leaves	Ding et al., 2017
*P. davidiana* × *P. bolleana*	Biomass production	*PtCYP85A3*	Seedlings	Jin et al., 2017
*P. simonii* × *P. nigra*	Salt and pathogen resistant	*PsnWRKY70*	Leaf disk	Zhao et al., 2017
*P. densiflora and P. trichocarpa*	Woody biomass	*PdGA20ox1/PtrMYB221*	Leaf disk	Cho et al., 2019
*P. deltoides* × *P. euramericana ‘Nanlin895’*	Drought resistance/salt tolerance	*DRS1*	Leaf disk	Kourosh et al., 2018
*P. alba* × *P. glandulosa*	Adventitious rooting	*PagFBL1*		Shu et al., 2019
*P. canescens*	Wood material improvement	*PCBER1*	Seedlings	Bruegmann et al., 2019
*P. euramericana* cv.	Drought resistance and salt tolerance	*PtHMGR*	Leaf disk	Wei et al., 2020
*P. tomentosa* Carr.	Trichome development	*miR319a; TCP19*	Leaf disk	Fan et al., 2020
*P. tomentosa*	Wood quality	*PtSS3*	Leaf disk	Li et al., 2020a
*P. alba* × *P. glandulosa*	Root development	*PtoWUSa*	Root	Li et al., 2020b
*P. alba* × *P. glandulosa*	Salt tolerance	*PtHDT902*	Stem	Ma et al., 2020
*P. Leucopyrami* -dalis 1	Low temperature stress	*Bp MBF1*	Leaf disk	Wang, 2020
*P. alba* × *P. glandulosa*	Root growth and drought resistance	*PdNF-YB21*	Leaf disk	Zhou Y. et al., 2020
*P. simonii* × *P. nigra*	Salt tolerance	*PsnHDZ63*	Leaf disk	Guo et al., 2021
*Betula platyphylla*	Abiotic Stress	*BpERF98*		Li et al., 2021a

Due to its simplicity and high repetition rate, leaf disk transformation is the most widely used method for plant transforming using *A*. *tumefaciens*. The transformation receptor, infection and coculture time, of genetic transformation might differ among forest tree species (Table 2). However, as explants, young leaves (top 3–5 leaves of tissue culture plantlets) are amenable to genetic transformation, and the infection and coculture times depend on the secondary metabolites produced by plants. Additionally, bacterial density in the logarithmic growth phase (optical density at 600 nm = 0.6–0.8) is suitable for the genetic transformation of most forest trees. Moreover, callus induction from plant organs and infection with *A*. *tumefaciens* can be conducted to generate transgenic plants. The stability of callus germination can be tested by using beta-glucuronidase (GUS)-labeled vectors to infect calli of *Hevea brasiliensis* (Lardet et al., 2011). A large number of transgenic plants can be produced in a relatively short period by *A*. *tumefaciens*-mediated transformation, contributing to the acquisition and rapid renewal of transgenic trees.

**TABLE 2:** Infection time and coculture time of different trees.

Tree species	Transformation receptor	Gene	Infection time	Co-culture time	References
*P. tomentosa*	Tissue culture seedling	Antisense *CCoAOMT*	15–20 min	2–3 days	Zhao et al., 2004
*Euonymus japonicus* ‘Cu Zhi’	Hypocotyl	*GAN*	40 min	3 days	Shang et al., 2008
*Juglans*	Somatic embryo		10–15 min	2 days	Wang, 2015
*P. trichocarpa*	Stem sections of 5–6-month-old trees		3–5 min	2 days	Wang, 2015
*P. tremula* × *P. alba*	Leaf disk		10 s–30 min	2–3 days	Bruegmann et al., 2019
*Eucommia ulmoides*	Leaf	*GUS*	10 min	3 days	Liu et al., 2020
*Lycium ruthenicum* Murr	Hypocotyl	*GUS*	5 min	2 days	Wang et al., 2020
*Populus leucopyrami*-dalis 1, L1	Leaf disk	*BpMBF1*	8–10 min	4 days	Wang, 2020
*P. simonii* × *P. nigra*	Leaf disk	*PsnHDZ63*	10 min	2–3 days	Guo et al., 2021
*Pyrus betulaefolia*	Seedlings	*mCherry*	3 h	18-45 d	Hao et al., 2021
*Betula luminifera*	Leaf	*GUS;GFP*	20 min	30 days	Liu et al., 2021
*Cunninghamia lanceolata*	Stem	*GUS*		3 days	Wei et al., 2021

*Agrobacterium tumefaciens*-mediated transformation is simple, economical, and efficient. It is also important for investigating gene function and the cultivation of transgenic plants. However, *A*. *tumefaciens* infection is limited to certain species and genotypes. *A*. *tumefaciens* residues may form crown galls, resulting in the yield reduction of transgenic plants (Guo et al., 2019). Further consideration should be given to the field applications of *A*. *tumefaciens*-mediated transgenic trees.

### Gene Gun-Mediated Transformation

The principle of the gene gun method is to use accelerators to transfer particles coated with exogenous genes into receptor cells, tissues, or organs so that the exogenous genes can be integrated into the receptor genome and expressed (Zhang et al., 2013). This method is mainly applied to crops and some fruit trees such as wheat, corn, bean, and citrus (Ozyigit and Yucebilgili Kurtoglu, 2020). Compared to *A*. *tumefaciens*-mediated transformation, the gene gun method is not limited by genotype (Ozyigit and Yucebilgili Kurtoglu, 2020). The applications of gene gun-mediated transformation in forest trees are listed in Table 3; gene gun technology has considerable potential in forest tree research.

**TABLE 3:** Gene gun-mediated transformation in different tree species.

Tree species	Characteristics	Gene	Transformation receptor	References
*P. nigra*	Insect resistant	*Bt*	Leaf	Li et al., 2000
*Citrus medica*	Selection marker	*GUS*	Leaf disk	Zhou et al., 2005
*P. euramericana* cl. ‘Bofeng 1’	Abiotic stress	*JERF36*; *SacB*; *ZxZF*; *GST*; *AREB*	Leaf disk	Cui, 2012

The efficiency of gene gun-mediated transformation is affected by receptor types, culture conditions, and transformation parameters (Wang B. et al., 2018). The transformation efficiency of plant cells or tissues with strong regeneration ability and strong physiological activity is high. For example, a highly efficient transformation system involving particle bombardment of the callus of date palm was reported (Mousavi et al., 2014). Gene gun transformation is also unrestricted in terms of the materials and cells to which it can be applied. The gene gun method could overcome the drawbacks of *A*. *tumefaciens*-mediated transformation and improve transformation efficiency. For example, the drought-related genes *JERF36*, *ZxZF*, *AREB*, and *GST* were cotransformed into *Populus euramericana* by particle bombardment to generate transgenic poplar with drought tolerance (Cui, 2012). However, gene gun-mediated transformation has low transformation efficiency, inserts multiple gene copies, and can inactivate or silence the transformed genes. Additionally, the exogenous genes are expressed unstably and easily lost on bombardment. There is an increased likelihood of non-transformants or chimerism, possibly leading to abnormal gene expression and coinhibition.

### Pollen Tube Pathway

The pollen tube pathway uses pollen tubes naturally formed after plant pollination to carry out genetic transformation and typically comprises three steps, namely, foreign gene injection into the pollen tube, integration into the plant genome, and selection of transgenic plants (Wang M. et al., 2018). Compared to other transformation methods, the pollen tube pathway undergoes a short period of application in transgenic plants, and there are few reports of its use in forest trees, so further research should be needed in this field. The pollen tube method was used to introduce the total DNA of *P*. *alba* into *Populus liaoningensis* sp. nov × *Populus deltoids* cv. “N001,” and the phenotypic characteristics of the donor poplar were evident in four transgenic lines (Zhao, 2016). Intriguingly, *Ve* gene transfer into the walnut genome by the pollen tube pathway resulted in a lower malformed fruit rate than stigma-cutting addition or microinjection (Hou et al., 2004). The method has been applied in crops, e.g., *Oryza sativa*, and *Glycine max*, but in few forest tree species (Guo and Zhou, 2018; Zhang H. et al., 2021; Table 4).

**TABLE 4:** Application of pollen tube passage method in forest trees.

Plant receptors	Characteristics	Gene	References
*Juglans regia* L.	Fruit setting rate	*Ve*	Hou et al., 2004
*Armeniaca vulgaris* Lam	Cold hardiness	*AFP*	Sun et al., 2005
*P. tomentosa* × *P. bolleana; P. alba* × *P. Tomentosa*; *P. alba* × *P. glandulosa*	Salt resistance	*P. euphratica* Oliv. DNA	Chen, 2008
*Juglans regia* L.	Fruit setting rate; herbicide resistance	*Bar*	Liu, 2012
*Populus* × *Liaoningensis* × N001 *P. deltoids* cv. ‘N001’	Character combination	*P. alba* DNA	Zhao, 2016

Although the pollen tube pathway is less frequently used than *A*. *tumefaciens*-mediated transformation, it overcomes the genotype restriction of the latter. For example, the genetic transformation of cotton is restricted by genotype, and transgenic cotton lines can be generated by the pollen tube pathway to enhance insect and herbicide resistance (Showalter et al., 2009). This has the advantage of simplicity and is also inexpensive but has low transformation efficiency (Zhang, 2010; Cui et al., 2013; Wang M. et al., 2018). However, the method is limited by flowering time and is not applicable to gymnosperms, as it is dependent on naturally formed pollen tubes (Jian et al., 2012).

### Protoplast Transformation

Genetic transformation of protoplasts refers to the transfer of exogenous genes into plants, using protoplasts as receptors to generate transgenic plants with stable expression of exogenous genes. Protoplasts, as single-cell systems, are not (or less) affected by the surrounding cells and microenvironment. Compared to protoplast transformation in annual herbaceous plants, such as *Arabidopsis thaliana*, tobacco, and *O*. *sativa* (Jiang et al., 2006; Sun et al., 2013; Zhao et al., 2014), the separation and regeneration of protoplasts in forest trees is difficult, although advances have been made (Table 5). For example, protoplasts were isolated from petals and leaves of *Camellia sinensis* (Liu et al., 2017; Peng et al., 2018; Ye et al., 2021); however, large-scale analysis is still under way. Intriguingly, the addition of aminophosphoric acid inhibitors degraded the cell wall, as verified in elm (Chang et al., 2018b). Additionally, green fluorescent protein (GFP) was transformed into *Elaeis guineensis* protoplast by a polyethylene glycol (PEG)-mediated method, and a protoplast transformation system of this species was established for the first time (Masani et al., 2014).

**TABLE 5:** Protoplast transformation in different tree species.

Tree species	Characteristics	Gene	Transformation receptor	References
*Elaeis guineensis*		*GFP*	Embryogenic cell	Masani et al., 2014
*P. davidiana* × *P. bolleana*	Insect resistant	*cry3Bb*	Leaf	Xu et al., 2020
*P. trichocarpa*	Subcellular localization	*BpFLA20*	Leaf	Yu et al., 2020
*Cunninghamia lanceolata*	Cells divide and regenerate	*GFP*	Secondary xylem	Wei et al., 2021
*Elaeis guineensis* Jacq.	Increase in conversion rate	*GFP; REP*	Leaf	Wang et al., 2021c

Protoplasts can be extracted from almost all organs and tissues and show intrinsic developmental and spatial characteristics. The regenerated plants develop from single-cell systems, which are easy to purify and stable. Therefore, the introduction of exogenous genes into protoplasts has advantages compared to other exosomes. PEG-mediated transformation, shock perforation transformation, liposome-mediated transformation, and *A*. *tumefaciens* coculture transformation are commonly used to construct protoplast-based genetic transformation systems (Zhao and Chen, 2004). The PEG-mediated method is the most widely used type of protoplast transformation and can be combined with the electroshock method to improve transformation efficiency (Lenaghan and Neal Stewart, 2019). Since there is no cell wall, this method overcomes the obstacles of poor hybrid compatibility and low cell-transformation efficiency. In addition, protoplasts can be isolated from uniform cell suspension cultures, mainly from calli. A system for protoplast regeneration to whole plants has been established in *A*. *thaliana*, which showed that *WUS* and *DRN* were necessary for protoplast regeneration and greatly facilitated this process (Xu et al., 2021). Cell-wall regeneration is a key step in protoplast regeneration to whole plants (Zhang Q. et al., 2021). Early screening of molecular targets by protoplasts enabled the establishment of efficient and automatic protoplast isolation, transformation, and screening methods in crops. However, protoplast separation and regeneration in forest trees are more difficult than in annual crops and have not been well-developed, hampering the development and application of protoplast transformation in forest trees.

### Instantaneous Transformation

Instantaneous genetic transformation enables the investigation of gene function and comparison of genetic constructs of recombined genes (Canto, 2016). Instantaneous transformation can be mediated by particle bombardment, PEG, plant virus vector, and *A*. *tumefaciens*. Due to the cost of particle bombardment equipment, the low success rate of protoplast culture, and scarcity of viral vectors, *A*. *tumefaciens*-mediated instantaneous transformation is typically used (Li et al., 2020c).

Leaf osmosis is the most commonly used instantaneous gene expression method in *A*. *tumefaciens* infection. For example, a method was established to reduce individual differences in the instantaneous transformation of *Camptotheca acuminata* (Wang B. et al., 2018). The transcription factor *LoNAC18* was transferred into larch by *A*. *tumefaciens* instantaneous transformation, demonstrating that *LoNAC18* is involved in the regulation of PEG-mediated simulated drought stress in larch (Zhang et al., 2020). Besides leaves, it could also be used in stems and roots. An instantaneous transformation system was established for vacuum osmotic infection of poplar stem segments, enabling identification of the functions of genes involved in vascular tissue differentiation and regulation of xylem development (Li et al., 2021b). Instantaneous transformation of roots has been applied in medicinal plants and soybeans, but there are few reports in forest trees (Meng et al., 2019; Xia et al., 2020; Table 6). Intriguingly, a simple and efficient *A*. *tumefaciens*-mediated instantaneous gene expression system was developed for diverse trees—including birch, poplar, and Tamarix—in which the whole plantlet, leaf, and stem are used as explants for instantaneous expression (Zheng et al., 2012). As genetic information on forest trees accumulates, the instantaneous transformation will enable the exploration of metabolic pathways and subcellular localization of forest tree genes. Therefore, considering the low transformation efficiency and non-availability of genetic transform systems, it is necessary to improve *A*. *tumefaciens*-mediated instantaneous transformation of forest trees for transgenic research.

**TABLE 6:** Application of instantaneous transformation in forest trees.

Tree species	Characteristics	Gene	Transformation receptor	References
*Rosa hybrida*	Flower development	*RhSAG*	Cutting seedlings	Wang et al., 2014
*Camptotheca acuminata*	Synthesis of camptothecin	*pBI121*	Seed	Wang B. et al., 2018
*P. alba* × *P. glandulosa*	Salt tolerance	*PdPTP1*	Leaf	Lu et al., 2020
*P.* .*alba* × *P. glandulosa*	Regulation mechanism of xylem development	*eYGFP*	Stem	Li et al., 2021b

Virus-induced gene silencing (VIGS) is a transcription suppression technique that facilitates the functional analysis of genes. VIGS has been applied in diverse plants, including herbs and fruit trees (Dommes et al., 2019) but few forest trees (Cui and Wang, 2017; Dommes et al., 2019). VIGS technology based on tobacco rattle virus (TRV) was successfully applied in *Populus euphratica*, *Populus canescens* (Shen et al., 2015), *H. brasiliensis* (Li et al., 2021c), and *Olea europaea* (Koudounas et al., 2020). Since VIGS can rapidly reduce the expression of target genes, it facilitates molecular function research in plants, including forest trees. Therefore, it is necessary to determine the optimal conditions for VIGS to silence target genes in forest trees, including the viral vector, ambient temperature, plant age or development stages, and inoculation method (Shi et al., 2021). Overall, VIGS enables gene function analysis of trees.

### Comparison of Transformation Methods

*Agrobacterium tumefaciens*-mediated transformation is affected by genotype and secondary metabolites. It is difficult to establish the *A*. *tumefaciens*-mediated genetic transformation system in some plants, but the method is important for investigating gene function in forest trees. In dicotyledonous plants, *A*. *tumefaciens*-mediated transformation is the first choice due to its high transformation efficiency. The gene gun method compensates for the genotype limitation of *A*. *tumefaciens*-mediated transformation. Additionally, gene gun-mediated transformation is important for research on gymnosperms such as *Pinus*, but its application is limited by cost. The pollen tube pathway and protoplast transformation methods may be preferred for some forest trees. Instantaneous transformation enables the establishment of stable genetic transformation systems and expression of the genes of *Populus*, *Pinus*, and other forest trees in tobacco or other easily transformed plants. For most investigations of gene function, *A*. *tumefaciens*-mediated transformation is used for multiple forest trees. However, consideration should be given to other methods, particularly transformation from scratch in forest trees because *A*. *tumefaciens* residues can lead to crown gall development and yield reduction (Stanton, 2018).

## Trait Improvement of Forest Trees

### Insect Resistance

Multiple insect-resistant genes—including *Bacillus thuringiensis* (*Bt*), protease inhibitor (*PI*), *Androctonus australis* hector insect toxin (*AaIT*), and chitinase genes—have been identified and applied in trees (Ren et al., 2021). Among them, *Bt* is the most widely used in insect resistance. Stable transfer of *Bt* into forest trees was first reported in transgenic poplar (McCown et al., 1991). Intriguingly, the simultaneous application of two *Bt* genes expanded the scope of insect resistance in transgenic forest trees (Wang et al., 2012; Dong et al., 2015). Consequently, means of enhancing forest tree resistance to insects by transforming two or more *Bt* genes warrant further research.

Overexpression of *PI* genes, including serine protease inhibitors (*SPIs*) and Kunitz trypsin inhibitor (*KTI*), resulted in insect death and prevented resistance development (Major and Constabel, 2008; Clemente et al., 2019). Bivalent resistance genes (*CryIAc* and *API*) were introduced into poplar, and the mortality rate of larvae was 60–80% (Li et al., 2007). In addition, genetic transformation with *PI* and *Bt* genes enhanced the insect resistance of transgenic plants. Transgenic poplar with *API* and dual *Bt* genes were toxic to Lepidoptera and Coleoptera and showed greater insect resistance than plants transformed with a single *Bt* gene (Wang G. et al., 2018).

The GM improvement of insect resistance has been realized in diverse forest trees, including *Populus* (Ren et al., 2021), *Eucalyptus* (Shao et al., 2002), *Picea* (Hammerbacher et al., 2014), *Ulmus* (Newhouse et al., 2007), *Pinus* (Grace et al., 2005), and *Tsuga* (Merkle et al., 2014). Transgenic forest trees were first used commercially in China (Chang et al., 2018a). Exogenous genes were expressed stably in 8- and 10-year-old transgenic poplar trees, and there was no significant developmental difference between 10-year-old transgenic and non-transgenic poplars (Ren et al., 2017). The current investigations suggested that the additive effect existed in transgenic forest trees with the same or different kinds of insect-resistant genes, which presented broad-spectrum insect resistance. The stability of exogenous insect resistance genes in transgenic forest trees was verified in 10-year-old transgenic poplars. However, the stability and effectiveness of insect resistance require validation in transgenic forest trees as perennials. Additionally, whether insects will develop tolerance warrants further investigation.

### Herbicide Resistance

It is necessary to control weeds during the early stages of tree growth. Mechanical herbicides are inefficient and costly and affect the normal growth and development of forest trees. Therefore, it is preferable to cultivate herbicide-resistant tree varieties. Bialaphos resistance (*bar*) is the most widely used herbicide resistance selective marker gene; it is derived from the soil bacterium *Streptomyces hygroscopicus* and induces resistance to phosphate-based broad-spectrum herbicides, such as Liberty Basta, and Finale (Lebedev et al., 2016). *Bar* has been inserted into diverse species and hybrids of *P*. *alba*, *Eucalyptus*, *Picea abies*, oak, and various conifers (Brukhin et al., 2000; Confalonieri et al., 2000; Harcourt et al., 2000; Bishop-Hurley et al., 2001; Zhang et al., 2005; Álvarez et al., 2009). These investigations indicated the broad application of *bar* in herbicide-resistant transgenic trees.

In addition, glutathione S-transferase (*GST*) genes encoding specific herbicide resistance to acetylchloroaniline were introduced into poplar hybrids, enhancing herbicide resistance (Gullner et al., 2001). The poplar clones INRA 353-38 (*Populus tremula* × *Populus tremuloides*) and 717-1B4 (*P*. *tremula* × *P*. *alba*) transformed with *bar*, and the male sterility gene *BARNASE* showed stable herbicide resistance within 8 years (Li et al., 2008). The selection of herbicide-resistant trees provides an alternative to non-chemical weed control. In future, gene-editing technology may be used to improve the herbicide resistance of forest trees.

### Disease Resistance

Disease resistance genes are mainly used in the molecular breeding of forest trees to improve plant antiviral and antibacterial defenses. Trichosanthin (*TCS*), a broad-spectrum antiviral gene, was transformed into *Paulownia* by *A*. *tumefaciens*-mediated method, and transgenic *Paulownia* lines with strong disease resistance were screened out (Liu et al., 2011). HbLFG1, a negative regulator of plant immunity, promoted infection by *Erysiphe quercicola* of *H. brasiliensis* (Li et al., 2021d). Poplar is threatened by *Melampsora* species, which cause poplar leaf rusts. Overexpression of *A*. *thaliana GALACTINOL SYNTHASE3* (At*GolS*) and *Cucumber sativus RAFFINOSE SYNTHASE* (Cs*RFS*) in hybrid poplar (*P*. *alba* × *Populus grandidentata*) increased susceptibility to *Melampsora aecidiodes* infection (La Mantia et al., 2018). Additionally, constitutive overexpression of *PtrWRKY18* and *PtrWRKY35* in poplar activated disease-related genes and increased the resistance of poplar to *Melampsora*, suggesting functional redundancy (Jiang et al., 2017). Besides, miRNA can promote plant disease resistance by participating in hormone signaling and regulating resistance (*R*) genes. In transgenic poplar, miR472a positively regulates resistance to *Colletotrichum gloeosporioides* infection by targeting *NBS-LRR* and negatively regulates resistance to *Cytospora chrysosperma* infection (Su et al., 2018). At present, there are many studies on miRNA and disease resistance in rice, potato, and other crops, but there are few reports in forest trees (Natarajan et al., 2018; Zhang et al., 2018). Further investigation of the roles of miRNAs in pathogen infection of trees is needed.

Studies of tree disease resistance and genetic engineering have promoted the breeding and improvement of tree varieties. Future studies should focus on the regulatory networks of tree responses to pathogens to reduce disease susceptibility.

### Resistance to Abiotic Stress

Plant abiotic stresses include cold, freezing, drought, salt, nutrient deficiency, and heavy metals (Gong et al., 2020). Investigation of gene function in response to abiotic stress could improve the environments of trees and so, expand their ranges in specific ecosystems and increase species richness (Xu and Zhai, 2021). Therefore, breeding new varieties of trees with strong resistance to stress is warranted.

Tree genetic engineering research has focused on salt and drought tolerance. Overexpression of *WOX11*/*12A* and *ThNAC12* in poplar increased salt tolerance, reactive oxygen species (ROS) scavenging, and the antioxidant enzyme activity of transgenic plants (Wang et al., 2021a,b). Interference with *FDL* expression enhanced the drought resistance of transgenic poplar (Yu et al., 2019). The K^+^/Na^+^ homeostasis of root cells and tolerance to salt stress were improved in transgenic poplar overexpressing *JERF36s* (Ding et al., 2020). These studies provided insight into the mechanism of salt tolerance improvement in plants and will facilitate breeding strategies to improve salt tolerance. The introduction of *BpMBF1* into poplar significantly improved cold resistance (Wang, 2020). Instantaneous overexpression of *JrGRAS2* in walnut enhanced the tolerance to high temperature (Yang et al., 2018). Overexpression of *PsnICE1* significantly enhanced the cold stress tolerance and antioxidant enzyme activity of transgenic poplars (Wang et al., 2021d).

Many other adverse environmental conditions also affect plant growth. The transcriptomic profiles of poplar under stresses suggest candidate genes of breeding (Yao et al., 2018; Chen et al., 2020), enabling investigation of plant regulatory networks. The correlations among stress response regulatory signals need further investigation.

### Wood Property Improvement

Wood structure and quality are critical traits for genetic improvement. Lignin content can be reduced by introducing genes that inhibit key enzymes in the lignin synthesis pathway. Downregulation of coumaroyl shikimate 3′-hydroxylase (*C3*′*H*), cinnamate 4-hydroxylase (*C4H*), and 4-coumarate-CoA ligase gene (*4CL*) reduces the lignin content in transgenic hybrid eucalyptus (*Eucalyptus urophylla* × *Eucalyptus grandis*) (Sykes et al., 2015). Cell-specific downregulation of *4CL* decreased the lignin content of transgenic poplars (Cao et al., 2020). In transgenic poplars with suppressed *C3H* and hydroxycinnamoyl transferase (*HCT*), the fiber cell diameter, vessel molecular diameter, and cell wall thickness were smaller, leading to decreased lignin content (Zhou et al., 2018). Therefore, suppression of lignin biosynthesis-related genes in transgenic forest trees decreased the lignin content, thus improving wood properties and biomass utilization.

Overexpression of *GAlactUronosylTransferase12* (*GAUT12*) in poplar increased xylan and galacturonic acid production and decreased growth (Biswal et al., 2018b). Accordingly, downregulation of *GAUT12* significantly improved saccharification efficiency and promoted the growth of transgenic poplars (Biswal et al., 2015). In addition, downregulation of *GAUT4* by RNA interference (RNAi) decreased the homogalacturonan (HG) and rhamnogalacturonan II (RG-II) contents and increased the biomass yield (Biswal et al., 2018a). Therefore, the *GAUT* gene family negatively regulates plant growth by regulating xylan biosynthesis. The suppression of *ACAULIS5* expression reduced the stem cytokinin level in hybrid aspen (*P*. *tremula* × *P*. *tremuloides*) and reduced secondary stem growth (Milhinhos et al., 2020). In *P*. *tomentosa*, PtSS3 is important in sucrose metabolism and growth and participates in wood formation (Li et al., 2020a). Brassinosteroid (BR) signaling plays an important role in secondary growth and wood formation. The BR signaling pathway affects xylem development synergetic with *PdC3H17*, a positive regulator of auxin-mediated xylem formation (Tang et al., 2020). However, to overcome the influence of the environment and obtain stable traits during the growth of transgenic plants, further improvement of the technology and accumulation of genes related to wood properties is needed.

### Flowering Regulation

Plants undergo the transition from infancy to reproductive maturity before flowering. Furthermore, trees experience a longer vegetative period than crops, prolonging the breeding cycle (Liao and An, 2013). However, genetic engineering can shorten infancy and alter flowering time in forest trees. FLOWERING LOCUS T (*FT*) is a floral hormone that affects plant flowering, growth, and development (Wigge, 2011). Overexpression of *FT*-induced flowering of *Eucalyptus*, and early flowering trees were found to be vigorous, showing a high branching phenotype (Klocko et al., 2016).

Transformation of poplars with HSP:*AtFT* and PsEND1:*barnase*-*barstar* vectors resulted in disturbed pollen development and the formation of male-sterile plants (Briones et al., 2020). Additionally, LEAFY (LFY) is necessary for the induction of flower organ-recognition genes. It endows root explant cells with the fate of flowers and allows callus to form flowers and flower organs without producing leaves (Wagner et al., 2004). In sweetgum, RNAi was used to inhibit LEAFY gene expression, generating sterile transgenic plants (Qiao et al., 2007). A vector with the RNAi-LFY cassette was transferred into *P*. *alba*, which markedly altered flower morphology and led to female flower sterility (Klocko et al., 2021). However, in asexual forest trees, sterility associated with *LFY* expression inhibition can alleviate the gene flow of seeds and pollen, although the effects on tree shape and wood production are unclear (Klocko et al., 2021). However, the use of *LFY* suppressor genes could be costly, and further research is needed.

### Clustered Regularly Interspaced Short Palindromic Repeats -Cas and RNA Interference Application

RNA-based approaches, including RNAi and CRISPR system, enable highly targeted modifications to enhance yield and stress resistance. These methods are typically based on *A*. *tumefaciens*-mediated genetic transformation. In RNAi, small interfering RNAs downregulate target gene expression without affecting the expression of other genes and are important for plant improvement (Rajput et al., 2021). GM agroforestry poplars obtained by RNAi exhibited reduced plantation isoprene emissions without compromising woody biomass production (Monson et al., 2020). Transgenic poplars carrying PTRARF2.1-RNAi showed severe leaf phenotypes, such as irregular shape and small size, and stimulated expression of auxin-response genes (Fu et al., 2019). In addition, RNAi allows the targeting of specific plant pathogens to control plant diseases (Kuo and Falk, 2020). By silencing *CYP33C9* by RNAi *in vitro*, the feeding, reproduction, oviposition, hatchability, and pathogenicity of *Bursaphelenchus xylophilus* nematode were inhibited (Qiu et al., 2019). The effects of RNAi should be studied and applied in other tree species.

The CRISPR system for precision breeding has been applied in poplar, *Eucalyptus*, and other forest tree species (Müller et al., 2020; Elorriaga et al., 2021). For example, the knockout of *CSE* by CRISPR-Cas9 improved lignocellulosic biomass without growth retardation in GM poplar (Jang et al., 2021). Knockout of the root growth transcription factor *PDNF*-*YB21* by CRISPR-Cas repressed the root growth and drought resistance in poplar (Zhou Y. et al., 2020). These studies aimed to improve sustainable production, induce DNA-free targeted mutations, and alter plant architecture, sex, and floral development. CRISPR-Cas technology does not introduce exogenous genes into the genomes of forest trees and so, has higher biosafety than other transgenic techniques. CRISPR-Cas is the most promising gene-editing technology developed to date (Bewg et al., 2018).

## Perspectives

**FIGURE 1:**
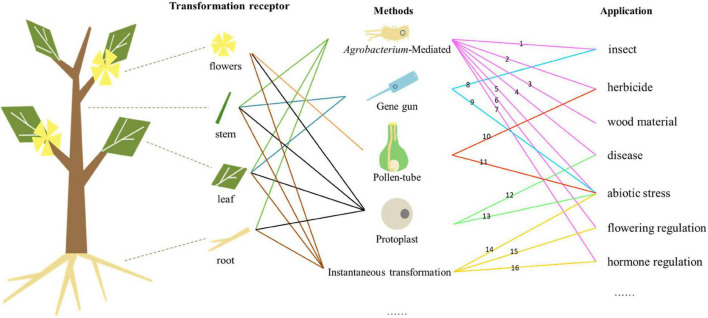
Relationships among genetic transformation receptors, methods, and applications. Numbers represent different gene types. (1) Bt, API, etc., (2) Bar, aroA, GST, etc., (3) 4CL, C3H, C4H, etc., (4) TCS, PPV, WRKY, etc., (5) MBF, BADH, etc., (6) FT, LFY, etc., (7) GAUT, BAK, RGL, etc., (8) Bt, etc., (9) GERF, AREB, etc., (10) Bar, etc., (11) AFP, etc., (12) hph, etc., (13) FLA, etc., (14) DREBRB, ALDH21, etc., (15) SAG, etc., and (16) pBI121, etc.

Genetic engineering can improve the traits of forest trees, shorten the breeding period, and enable the cultivation of new varieties with high commercial value by introducing exogenous genes (Figure 1). Genetic transformation also enables the exploration of gene function in forest trees. However, there are many difficulties and problems to overcome in forest trees. One of them is the genetic transformation of vectors with multiple foreign genes. The introduction of multiple exogenous genes concurrently could improve the traits of forest trees, but the construction of vectors carrying multiple genes is more difficult and some may not play the expected roles in transgenic trees. For example, transgenic poplar with two insect-resistance genes (*Cry1Ac* and *Cry3A*) and two salt-tolerant genes (*mtlD* and *BADH*) did not show improved salt tolerance (Zhou X. et al., 2020). Additionally, the balance between the expression of exogenous genes and growth/development requires investigation—whether increased resistance weakens other traits in transgenic forest trees is unclear.

Due to the uncertainty over insertion sites, genetic transformation inevitably generates chimerism, for instance, in stems and young leaves. For example, in peach (*Prunus persica*), *A*. *tumefaciens*-mediated transformation is inefficient, with a low level of correspondence between transformed and regenerative cells, and a high rate of chimerism in the buds produced during transformation (Ricci et al., 2020). Although chimeras enhance the cultivation of some ornamental plants, the purification and stable inheritance of target types can be problematic. Conversely, the protoplast is a single-cell system that can develop into a complete plant, enabling stable inheritance of traits. Several genes enhance protoplast regeneration, in particular, callus formation, which might promote the use of protoplast-mediated gene transformation (Xu et al., 2021). A strict screening system is needed for protoplast transformation, identifying transgenic plants with improvements in the desired traits.

Genetic engineering is controversial due to the potential for harm to the environment. The long-term stability of transgenic forest trees needs to be investigated, and the environmental impact of GM trees is still debated. A 5-year field trial showed no effect of *Bt* transgenic 741 poplar on arthropods or soil bacterial diversity (Zuo et al., 2018). A robust biosafety framework is necessary, with precautions followed for domesticated trees. An international group of researchers in silviculture, forest tree breeding, forest biotechnology, and environmental risk assessment examined how the environmental risk assessment paradigm used for genetic engineering crop plants could be applied to the genetic engineering of trees for plantation. It is also important to differentiate between environmental risk assessment for confined field trials of genetic engineering trees and unconfined or commercial-scale release (Häggman et al., 2013).

It is important to establish a rapid and reliable transformation system for forest trees, considering their long growth cycle and low transformation efficiency. Multi-omics techniques and modern biotechnology will facilitate the molecular breeding of forest trees. Transgenic research on trees will improve transformation efficiency and enable the safety evaluation of transgenic plants for commercial application. Leveraging the genetic transformation of forest trees for ecosystem restoration, energy supply, and sustainable production is a major challenge.

## Author Contributions

YY and CW were involved in planning and drafting the manuscript. DX modified the manuscript. YL collated the table contents. YW conceived of the presented idea and supervised this study. All authors discussed the results and commented on the manuscript.

## Conflict of Interest

The authors declare that the research was conducted in the absence of any commercial or financial relationships that could be construed as a potential conflict of interest.

## Publisher’s Note

All claims expressed in this article are solely those of the authors and do not necessarily represent those of their affiliated organizations, or those of the publisher, the editors and the reviewers. Any product that may be evaluated in this article, or claim that may be made by its manufacturer, is not guaranteed or endorsed by the publisher.
